# The diagnostic role of DNA methylation in sporadic endometrial cancer: a systematic review and meta-analysis

**DOI:** 10.18632/oncotarget.23480

**Published:** 2017-12-20

**Authors:** Yu Fan, Yu Wang, Shaozhi Fu, Linglin Yang, Sheng Lin, Qingze Fan, Qinglian Wen

**Affiliations:** ^1^ The Department of Oncology, The Affiliated Hospital of Southwest Medical University, Luzhou City, Sichuan Province, P.R.China; ^2^ The Department of Health Examination, The Affiliated Hospital of Southwest Medical University, Luzhou City, Sichuan Province, P.R.China; ^3^ The Department of Pharmacy, The Affiliated Hospital of Southwest Medical University, Luzhou City, Sichuan Province, P.R.China

**Keywords:** DNA methylation, endometrial cancer, biomarker, diagnosis, meta-analysis

## Abstract

**Background:**

Although increasing numbers of methylated genes have been identified as biomarkers for endometrial cancer, the results have been inconsistent. We therefore carried out a systematic review and meta-analysis to evaluate the diagnostic accuracy of methylated genes as markers for sporadic endometrial cancer.

**Results:**

A total of 22 studies including 1930 participants (sporadic endometrial cancer patients and normal individuals) met our eligibility criteria. The pooled sensitivity and specificity were 0.93 (95% confidence interval: 0.91−0.94) and 0.48 (95% confidence interval: 0.46–0.50), respectively. The area under the summary receiver operating characteristic curve was 0.8834. The presence of DNA methylation was significantly associated with lymph node metastasis of endometrial cancer (pooled odds ratio: 0.28, 95% confidence interval: 0.15–0.52, *p* < 0.001).

**Materials and Methods:**

We searched the relevant literature systematically using the PubMed and Web of Science databases up to April 2017. Diagnostic accuracy variables were pooled and analyzed using Meta-DiSc software. Sensitivity analysis and publication bias were evaluated using Review Manager.

**Conclusions:**

This meta-analysis suggests that the detection of DNA methylation is associated with lymph node metastasis, with high sensitivity but relatively low specificity for the diagnosis of sporadic endometrial cancer.

## INTRODUCTION

Endometrial cancer (EC) is one of the three main tumors originating in the female genital system. Its incidence is higher than that of cervical cancer in many countries, and is located in the top of gynecologic malignant tumors [1, 2]. EC patients often present with abnormal vaginal bleeding; although many patients are diagnosed when the disease is still confined to the uterus, about 30% are diagnosed with EC in its later stage. Most clinical trials for the treatment of advanced and recurrent EC have shown limited benefits, and the mortality rate has increased dramatically over the past few years [3, 4].

Abnormal genetic and epigenetic alterations have been widely recognized as being associated with EC [5, 6]. However, genetic markers have not yet proven reliable for identifying the entire spectrum of the disease, especially sporadic EC. Aberrant DNA methylation is one of the most widely studied epigenetic modifications with a critical role in EC [7, 8]. Diagnostic markers based on gene methylation have recently been developed and have shown considerable promise for the detection of EC, while aberrant promoter methylation has been found to be an early and widespread alteration in endometrial tumorigenesis [9]. However, although specific gene methylation patterns have been widely used for the diagnosis of many different cancers, including EC, there is currently a lack of effective diagnostic biomarkers for EC, and novel, accurate markers are urgently needed.

Previous studies have investigated the use of aberrant gene methylation in tissue samples as potential diagnostic biomarkers for EC, with encouraging but variable results. Further studies are therefore needed to describe the associations between DNA methylation in different tumor suppressor genes and the clinicopathologic features of sporadic EC. Moreover, no systematic review or meta-analysis has yet been conducted to evaluate the diagnostic accuracy of the existing studies. We therefore conducted a comprehensive, systematic review and meta-analysis of eligible studies to resolve the inconsistent and ambiguous findings, and to clarify the diagnostic value of DNA methylation in EC. Furthermore, we aimed to identify the diagnostic accuracy of gene methylation markers to predict other clinical pathological outcomes of EC.

## RESULTS

### Study characteristics

**Figure 1:**
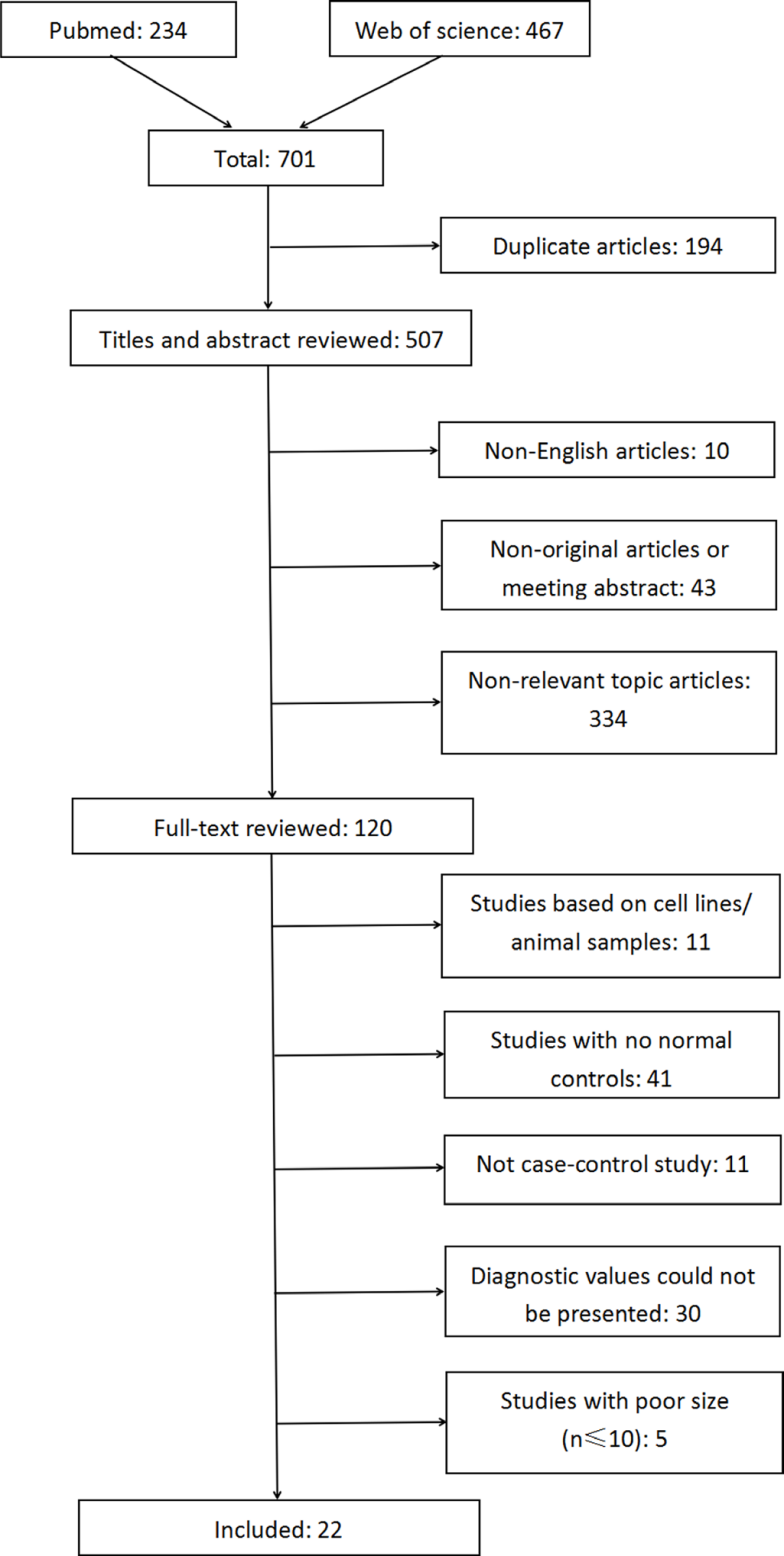
Flow diagram of the literature search process (search prior to 30/04/2017)

The workflow of the systematic literature search is displayed in Figure 1. The primary search of PubMed and Web of Science identified 701 articles, of which 194 were duplicate articles. A total of 120 studies were initially obtained after filtering the titles, abstracts, and full texts. Of these, 98 studies were excluded based on the eligibility criteria: 11 studies were excluded based on the use of *in vitro*/*ex vivo* cell lines and human xenografts; 41 studies did not have healthy normal controls; 11 were not case-control studies; it was not possible to extract or calculate the diagnostic sensitivity and specificity of the methylation biomarkers in 30 studies; and five studies had small sample sizes (*n* ≤ 10). All the included studies focused on DNA methylation/hypermethylation in tissues. The systematic literature search thus finally yielded 22 studies including 1930 participants (1418 patients and 512 normal individuals). None of the patients had received preoperative chemotherapy, radiotherapy, or hormone therapy. The included studies were published between 2001 and 2016 and originated from nine regions (China, Czech Republic, Hong Kong, Japan, Netherland, Russia, Slovak Republic, Taiwan, and USA) (Table 1). The sample sizes of these studies ranged from 19–155 (median 88). Fourteen studies evaluated the diagnostic value of methylation of a single gene (Sasaki et al., 2001(1) [10]; Sasaki et al., 2001(2) [11]; Saito et al., 2003 [12]; Sasaki et al., 2003 [13]; Li et al., 2005 [14]; Pijnenborg et al., 2007 [17]; Yanokura et al., 2007 [18]; Tse et al., 2009 [20]; Varley et al., 2009 [21]; Yi et al., 2011 [22]; Kovalenko et al., 2013 [25]; Yang et al., 2013 [27]; Chmelarova et al., 2014 [28]; Dong et al., 2015 [30]), five studies evaluated multiple genes (Banno et al., 2006 [15]; Suehiro et al., 2008 [19]; Fiolka et al., 2013 [24]; Visnovsky et al., 2013 [26]; Sheng et al., 2016 [31]), and the other three studies evaluated both single and combined genes (Shih et al., 2006 [16]; Zhang et al., 2011 [23]; Chen et al., 2015 [29]). Seventeen studies measured the methylation patterns of the genes using methylation-specific polymerase chain reaction (MSP), three studies used combined bisulfite restriction analysis (COBRA), one used quantitative MSP (qMSP), and one used MSP and COBRA for two genes, respectively. Details of the DNA methylation biomarkers and their diagnostic powers are shown in Supplementary Table 1. All 22 selected publications were evaluated and checked by two reviewers. High levels of methodological quality (more than five stars) were observed according to the NOS scale.

**Table 1:** The major characteristics of all included studies

No.	Study	Region	Method	Biomarker	Case	Control	Alteration type
1	Sasaki et al. (2001) [10]	Japan	MSP	single	83	33	hypermethylation
2	Sasaki et al. (2001) [11]	Japan	MSP	single	88	46	methylation
3	Saito et al. (2003) [12]	Japan	MSP	single	104	21	hypermethylation
4	Sasaki et al. (2003) [13]	Japan	MSP	single	60	10	methylation
5	Li et al. (2005) [14]	Japan	MSP	single	64	16	hypermethylation
6	Banno et al. (2006) [15]	Japan	MSP	multiple	52	18	methylation
7	Shih et al. (2006) [16]	Taiwan	MSP	single/combined	35	20	methylation
8	pijnenborg et al. (2007) [17]	Netherland	MSP	single	95	27	methylation
9	Yanokura et al. (2007) [18]	Japan	MSP	single	50	9	hypermethylation
10	Suehiro et al. (2008) [19]	Japan	MSP/COBRA	multiple	106	27	hypermethylation
11	Tse et al. (2009) [20]	Hongkong	COBRA	single	125	30	methylation
12	Varley et al. (2009) [21]	USA	COBRA	single	14	5	methylation
13	Yi et al. (2011) [22]	China	MSP	single	82	32	methylation
14	Zhang et al. (2011) [23]	China	MSP	single/combined	35	22	methylation
15	Fiolka et al. (2013) [24]	Slovak	MSP	multiple	41	20	methylation
16	Kovalenko et al. (2013) [25]	Russia	COBRA	single	18	10	methylation
17	Visnovsky et al. (2013) [26]	Slovak	MSP	multiple	50	35	methylation
18	Yang et al. (2013) [27]	China	MSP	single	97	40	methylation
19	Chmelarova et al. (2014) [28]	Czech	MSP	single	54	18	methylation
20	Chen et al. (2015) [29]	Taiwan	qMSP	single/combined	26	18	methylation
21	Dong et al. (2015) [30]	China	MSP	single	80	28	hypermethylation
22	Sheng et al. (2016) [31]	China	MSP	multiple	59	27	methylation

### Meta-analysis of diagnostic value

**Figure 2:**
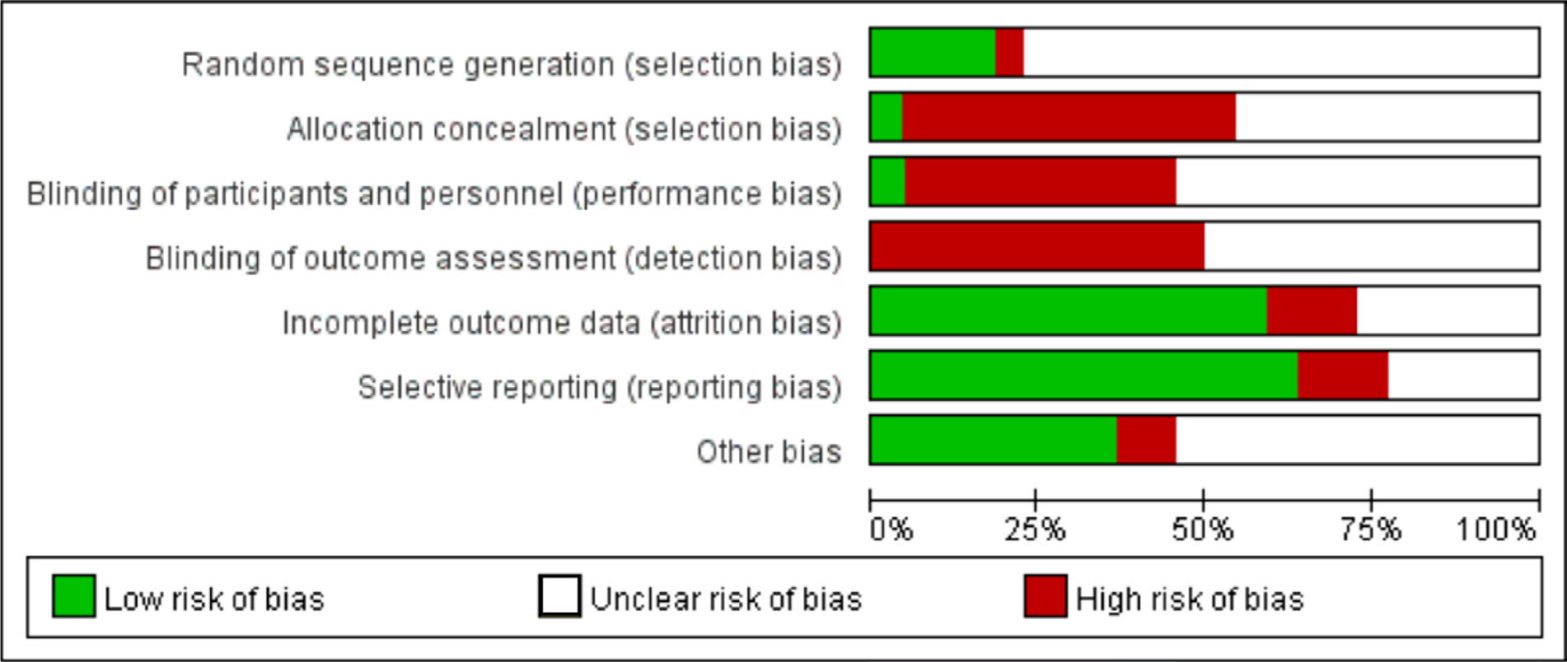
Risk of bias graph (reviewers’ judgements about each risk of bias item presented as percentages across enrolled studies)

We assessed the risk of bias for each study. The detailed evaluation criteria and results for each item are shown in Supplementary Table 2 and Supplementary Figure 1, and the risk of bias is summarized in Figure 2. The risk of bias was high or unclear in most included studies. Four studies stated that the sequences of participants were generated randomly. The diagnostic values for all the assessed methylation biomarkers were reported in 64% of studies, indicating no selective reporting. Eight studies were reported to be free of other biases and were defined as low risk.

**Figure 3:**
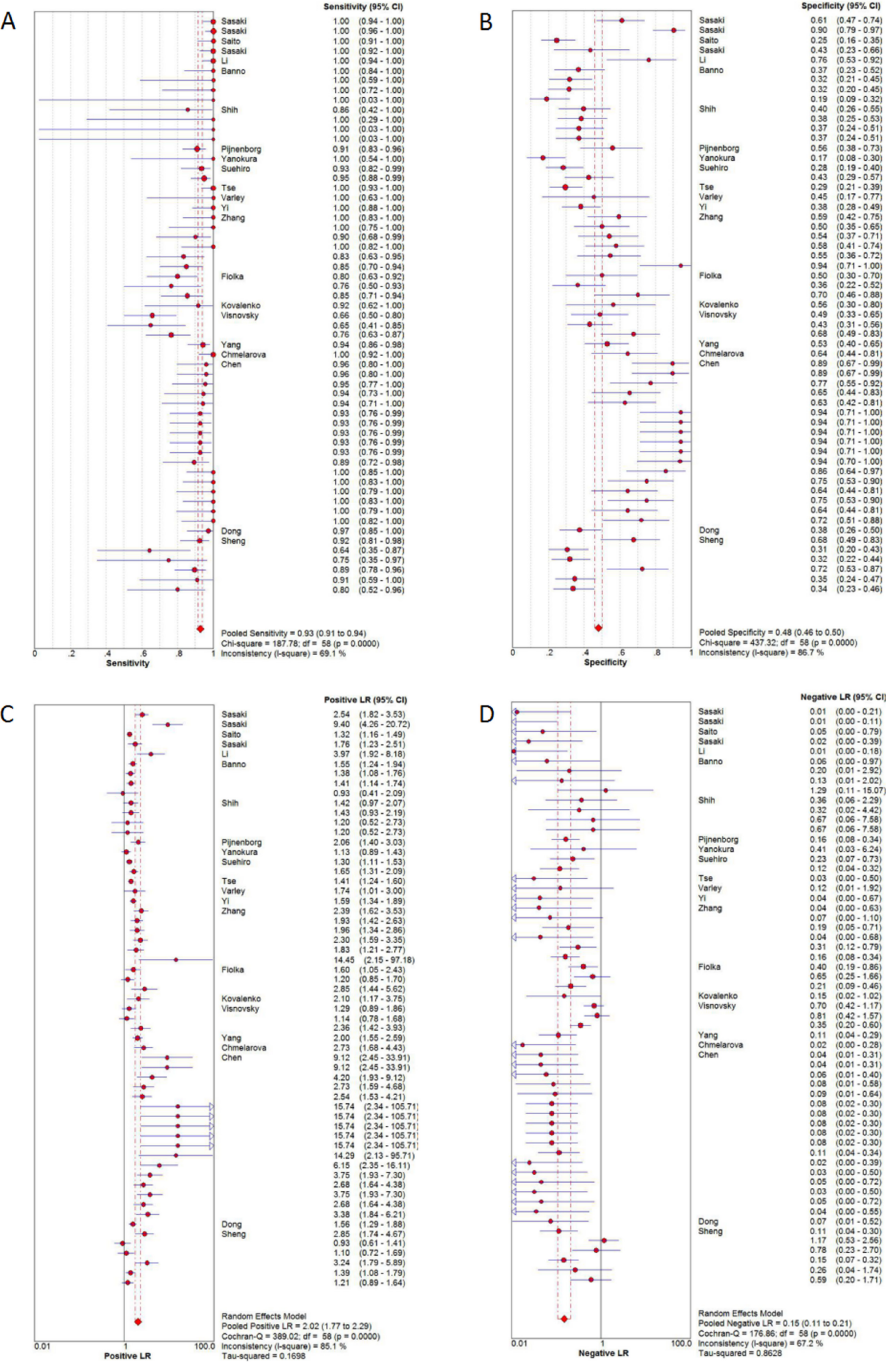
Diagnostic accuracy of forest plots (**A**) Forest plots of pooled sensitivity. (**B**) Forest plots of pooled specificity. (**C**) Forest plots of pooled positive likelihood ratio. (**D**) Forest plots of pooled negative likelihood ratio.

Twenty-two studies were pooled for the meta-analysis of diagnostic accuracy. The pooled sensitivity and specificity were 0.93 (95% confidence interval [CI]: 0.91–0.94) and 0.48 (95% CI: 0.46–0.50), respectively (Figure 3). The PLR and NLR were 2.02 (95%CI: 1.77–2.29) and 0.15 (95% CI: 0.11–0.21), respectively, and the pooled DOR was 18.84 (95% CI: 12.01–29.54). Significant heterogeneity was observed in the diagnostic meta-analysis of these studies (sensitivity: *I*^2^ = 69.1%, *p* < 0.001; specificity: *I*^2^ = 86.7%, *p* < 0.001). There was no significant threshold effect according to Spearman correlation (*ρ* =−0.19, *p* = 0.15). Subgroup analysis (Table 2) was therefore carried out according to five different parameters: sample size (<85 vs. ≥85), alteration type (methylation vs. hypermethylation), race (Mongolian vs. Caucasian), detection methods (MSP vs. COBRA vs. qMSP), and genes (single vs. any one vs. both genes). Lower heterogeneities for sensitivity were only detected in the subgroups of COBRA (sensitivity: *I*^2^ = 44.8%, *p* = 0.143), qMSP (sensitivity: *I*^2^ = 0%, *p* = 0.547), any one gene (sensitivity: *I*^2^ = 0%, *p* = 0.920), and both genes (sensitivity: *I*^2^ = 0%, *p* = 1.0); while the specificity heterogeneity was reduced in the Caucasian (specificity: *I*^2^ = 39.8%, *p* = 0.092) and any one gene (specificity: *I*^2^ = 0%, *p* = 1.0) subgroup. Subgroup analysis for DOR revealed homologous trends in hypermethylation, COBRA, qMSP, and one or two genes (hypermethylation: *I*^2^ = 49.9%, *p* = 0.062; COBRA: *I*^2^ = 0%, *p* = 0.871; qMSP: *I*^2^ = 0%, *p* = 0.994; any one gene: *I*^2^ = 0%, *p* = 0.998; both genes: *I*^2^ = 32.6%, *p* = 0.157). These results suggested that the methylation-detection method and type of gene combination might contribute to the heterogeneity. The other measures of diagnostic value in the subgroups are summarized in Table 2. Meta-regression analysis based on those factors was also applied to explore the possible heterogeneity source, as shown in Supplementary Table 3. Only the detection method significantly changed the heterogeneity of the universal diagnostic value (*p* < 0.001). Taken together, we considered that the detection method and gene combination contributed to the heterogeneity source. Further, well-designed studies with different detection methods are therefore needed to clarify the diagnostic role of gene methylation in sporadic EC.

**Table 2:** Subgroup analysis of diagnosis parameters

Subgroup	Sensitivity			Specificity			Diagnostic odds ratios		
	Value	*I*^2^ (%)	*P*	Value	*I*^2^ (%)	*P*	Value	*I*^2^ (%)	*P*
**Size**									
<85	0.94 (0.92–0.96)	47.5	0.001	0.53 (0.51–0.56)	85.8	0.000	29.08 (16.79–50.36)	53.3	0.000
≥85	0.91 (0.89–0.93)	83.4	0.000	0.42 (0.40–0.45)	86.4	0.000	9.18 (4.64–18.17)	76.8	0.000
**AT**									
methylation	0.92 (0.90–0.93)	67.7	0.000	0.51 (0.48–0.53)	86.0	0.000	18.73 (11.46–30.59)	70.1	0.000
hyper.	0.98 (0.95–0.99)	51.0	0.057	0.36 (0.32–0.41)	86.9	0.000	19.79 (6.64–58.99)	49.9	0.062
**Race**									
Mongolian	0.95 (0.94–0.96)	52.1	0.000	0.48 (0.45–0.50)	88.6	0.000	25.50 (15.43–42.15)	61.6	0.000
Caucasian	0.83 (0.79–0.87)	76.4	0.000	0.52 (0.46–0.57)	39.8	0.092	5.52 (2.72–11.17)	65.3	0.002
**Method**									
MSP	0.91 (0.90–0.93)	76.2	0.000	0.43 (0.41–0.45)	83.1	0.000	9.41 (5.72–15.49)	66.8	0.000
COBRA	0.97 (0.93–0.99)	44.8	0.143	0.37 (0.30–0.44)	50.6	0.108	15.84 (6.34–39.56)	0.0	0.871
qMSP	0.95 (0.93–0.97)	0.0	0.547	0.79 (0.75–0.83)	54.7	0.004	110.07 (59.44–203.82)	0.0	0.994
**Gene target**									
single	0.92 (0.91–0.94)	74.8	0.000	0.44 (0.42–0.47)	83.9	0.000	12.40 (7.74–19.87)	67.3	0.000
any one	0.91 (0.86–0.94)	0.0	0.920	0.94 (0.88–0.98)	0.0	1.000	162.57 (65.25–405.07)	0.0	0.998
both genes	1.00 (0.97–1.00)	0.0	1.000	0.54 (0.49–0.60)	81.0	0.000	31.68 (9.31–107.76)	32.6	0.157

Abbreviation: AT = alteration type; *I*^2^ = inconsistency index; hyper. = hypermethylation; *P* = *p* value.

### Association of DNA methylation with clinicopathological characteristics in patients with sporadic EC

We evaluated the association between DNA methylation and several clinicopathological features in patients with sporadic EC based on 13 different studies, including three involving multiple genes. We did not analyze age in relation to DNA methylation because there was no single cut-off value for age among the patients. Methylation status was significantly associated with lymph node metastasis (negative/positive, pooled odds ratio [OR]: 0.28, 95% CI: 0.15–0.52, *p <* 0.001) (Table 3), indicating that gene methylation was positively associated with the risk of lymph node metastasis in patients with sporadic EC, and thus potentially with a relatively poor prognosis. However, gene methylation status showed no significant relationship with body mass index (≤25.9/>25.9, pooled OR: 1.04, 95% CI: 0.44–2.47, *p* = 0.92), pathological type (endometrioid/other, pooled OR: 0.63, 95% CI: 0.21–1.88, *p* = 0.41), grade (G1/G2–3, pooled OR: 0.80, 95% CI: 0.41–1.54, *p* = 0.50), invasion (<1/2/≥1/2, pooled OR: 0.58, 95% CI: 0.19–1.76, *p* = 0.33), and stage (I–II/III–IV, pooled OR: −0.08, 95%CI: −0.20–0.04, *p* = 0.20).

**Table 3:** Meta-analysis of the association DNA methylation with clinicopathological features

Stratification	No. of studies	No. of patients	Pooled OR	95% CI	*P*	Heterogeneity	
						*I*^2^ (%)	*P*
BMI (≤25.9/>25.9)	5	130	1.04	0.44–2.47	0.92	0.0	0.50
Pathological type (endometrioid/others)	3	228	0.63	0.21–1.88	0.41	0.0	0.95
Grade (G1/G2-3)	18	877	0.80	0.41–1.54	0.50	65.0	<0.001
Invasion (<1/2/≥1/2)	6	413	0.58	0.19–1.76	0.33	82.0	<0.001
Lymph metastasis (negative/positive)	6	384	0.28	0.15–0.52	<0.001	0.0	0.85
Stage (I-II/III-IV)	16	789	-0.08	–0.20–0.04	0.20	72.0	<0.001

Abbreviation: The implication of No. of studies and patients containing multiple and different genes detected in the same study; *P = p* value; OR=odds ratio; *I*^2^ = inconsistency index; 95% CI = 95% confidence intervals; BMI = body mass index; others = non-endometrioid; Invasion = myometrial invasion.

### Sensitivity analysis and publication bias

**Figure 4:**
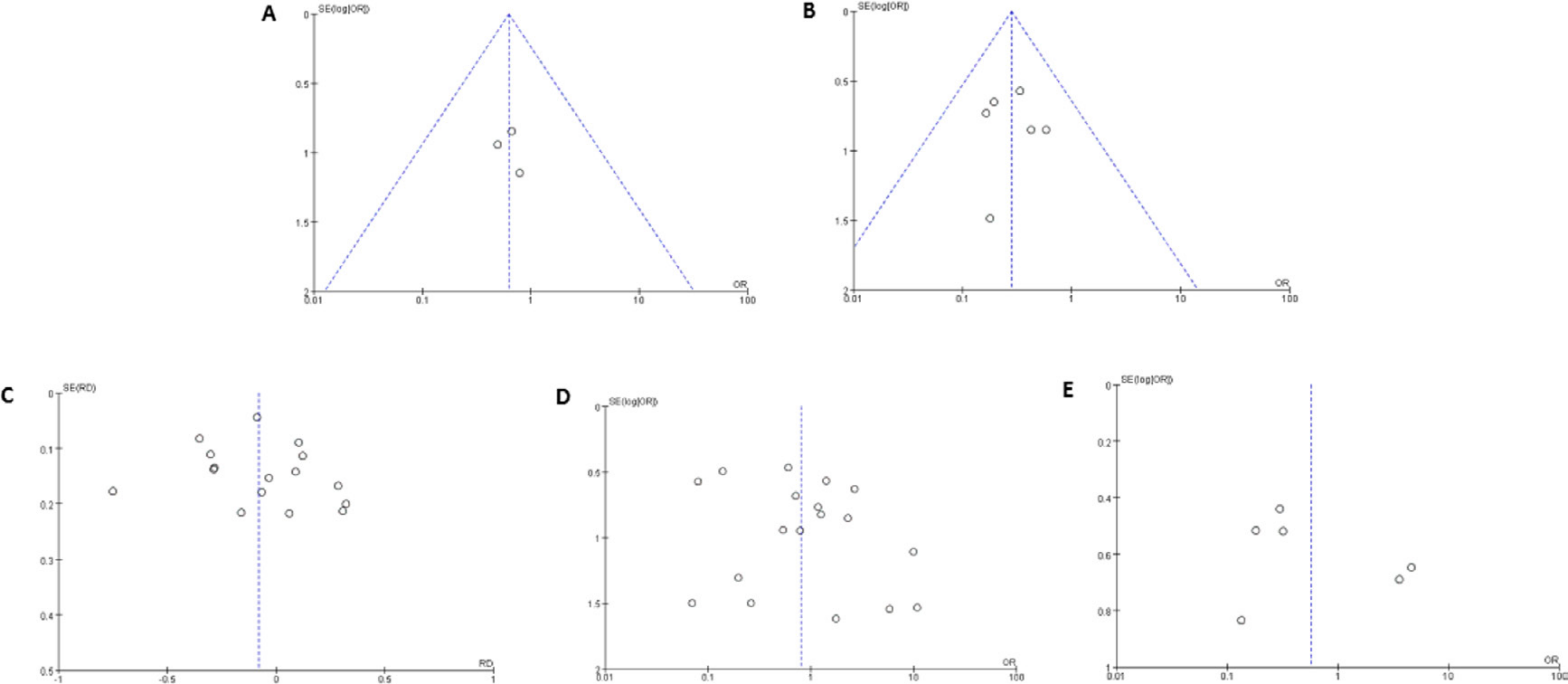
Funnel plots for the evaluation of publication bias The funnel plots from 13 enrolled studies (including 20 different genes) comparing endometrioid and non-endometrioid EC (**A**), comparing negative and positive lymph metastasis EC (**B**), comparing early staged (I-II) and advanced EC (III-IV) (**C**), comparing grade 1 and grade 2-3 EC (**D**), and comparing EC with myometrial invasion <1/2 and ≥1/2 (**E**). X-axis:value of odds ratio (OR); Y-axis: standard errors (SE) multiply log scale of OR.

Sensitivity analysis was conducted to evaluate the stability of the results, by removing one study at a time. However, this did not significantly affect the pooled OR or inconsistency index, indicating the stability of our meta-analysis. The funnel plot was relatively symmetrical and no single study fell outside the funnel (Figure 4), suggesting that there were no significant publication biases in this meta-analysis of gene methylation in patients with sporadic EC.

## DISCUSSION

Surgical resection or biopsy specimens are used for diagnosing numerous cancers, and are considered to be the gold standard for clinical applications. Pathological results from these tissues can also provide important information in relation to clinical decision making. DNA methylation has previously demonstrated potential use as a biomarker for EC, showing distinctly different patterns between EC and normal tissues [32]. Alterations in DNA methylation in EC reflect transcription changes during endometrial carcinogenesis. According to a recent review, numerous studies have assessed abnormal promoter methylation of different genes in EC [33]. Although methylation studies in relation to EC remain at the preclinical stage, many results offer the potential for clinical use as diagnostic, prognostic, and therapeutic-response biomarkers, as well as targets of epigenetic therapies. However, although aberrant methylated regions of *P16* and *RASSF1A* demonstrated sensitivity and specificity for the detection of EC [34, 35], aberrant methylation statuses of other genes, such as *ER*, *PR*, *MLH1*, *MGMT*, *APC*, and *CDH1*, have shown variable diagnostic accuracies for EC [33].

**Figure 5:**
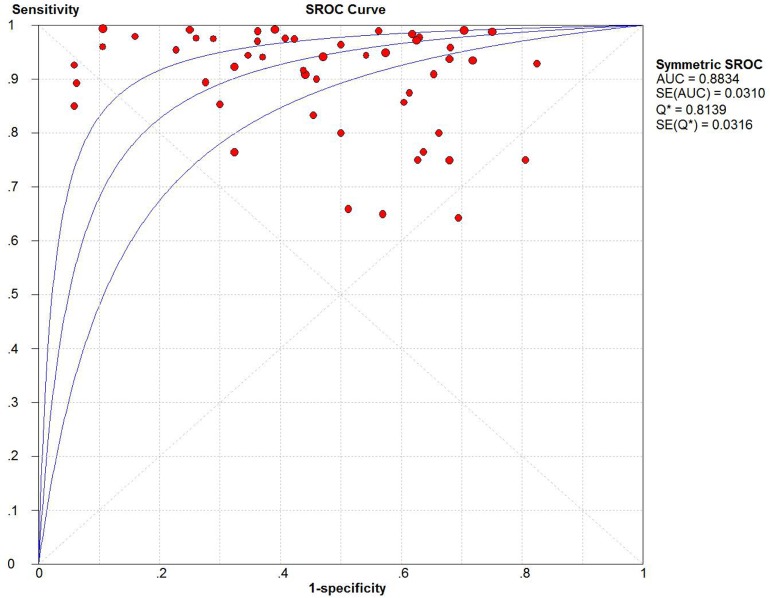
Summary receiver operating characteristic (SROC) plot with the associated 95% confidence region

In the present study, we evaluated the diagnostic accuracy of methylated markers for sporadic EC, based on previously published studies. Overall, the pooled sensitivity and specificity of DNA methylation for EC diagnosis were 0.93 and 0.48, respectively, and the area under the SROC curve (AUSROC) was 0.88 in tissue samples. The presence of DNA methylation had a relatively high diagnostic ability (area under curve [AUC] = 0.8834) for the risk of sporadic EC (Figure 5), but a relatively low specificity (0.48) and high sensitivity (0.93). In terms of sensitivity, DNA methylation is chemically stable and has shown high sensitivity in other tumors [36]. The present evidence demonstrated that methylated genes were superior to protein biomarkers such as CA-125, either alone or in combination, which had diagnostic sensitivities of only 40%–80% [37, 38]. A clinical diagnosis of EC currently relies on a combination of ultrasound, magnetic resonance imaging, and CA-125, though none of these alone is completely satisfactory. CA-125 is often used as a biomarker for ovarian cancer, but has also been suggested as a good prognostic marker for EC [37]. The presence of circulating tumor DNA (ctDNA) has been considered to be highly specific for certain cancers, mainly because somatic mutations identified in tumor DNA are absent from normal DNA, whereas gene methylation may occur in normal as well as cancer DNA [39]. Our study confirmed that the detection of DNA methylation in clinical samples had a relatively low diagnostic specificity for EC (pooled specificity: 0.48, 95%CI: 0.46–0.50), suggesting that more specific methylated genes are required.

The current study included gene methylation or hypermethylation alterations, which could lead to aberrant silencing of tumor suppressor genes in most cases. Consistent with these types of gene alterations, the most common detection method for methylation was MSP, which has various advantages including relatively high sensitivity [40]. In our meta-analysis, *MLH1*, *CDH1*, and *RASSF1A* gene methylation were detected in more than one study. *MLH1* was identified as the cause of abnormal DNA mismatch repair and microsatellite instability (MSI), and its dysfunction might be associated with Lynch syndrome (also known as hereditary nonpolyposis colorectal cancer syndrome, HNPCC) [41]. Although MSI has been detected in numerous patients with EC, its mutation frequency is extremely low, suggesting that it may arise as a result of aberrant methylation of promoter regions in MSI-positive EC patients [15]. Furthermore, the diagnostic value of aberrant DNA methylation for EC was not significantly influenced by the technique used, except possibly for qMSP, suggesting that this kind of assay may show greater specificity. A combination of gene methylation markers also showed potentially higher diagnostic accuracy in sporadic EC.

This preliminary study clearly indicated that detecting gene methylation patterns could offer a diagnostic test for sporadic EC with high sensitivity, but low specificity. The presence of DNA methylation in EC patients may predict positive lymph node metastasis and unfavorable survival. In general, there is lack of diagnostic and prognostic biomarkers for EC, and methylation markers may thus be useful for distinguishing between EC and non-malignant causes in women with abnormal vaginal bleeding. Further meta-analyses need to be conducted to address the use of accurate methylated targets and suitable detection techniques, while more prospective studies utilizing consistent and standardized methodologies are urgently required to resolve these problems.

This study had several limitations. Firstly, selection bias might have occurred due to enrichment of studies reporting positive results, and the relatively small sample sizes in some of the selected literature may also have led to bias. Furthermore, the included studies were mostly from East Asia, and the conclusions may therefore not be universally applicable. The use of different MSP primers and/or equipment, and the lack of a well-accepted methylated gene in sporadic EC might also have been potential sources of bias.

## MATERIALS AND METHODS

### Search strategy

We conducted a systematic literature search to identify studies assessing DNA methylation changes as biomarkers for the early diagnosis of sporadic EC. We searched the PubMed and Web of Science databases for all relevant English-language papers published prior to April 30, 2017, using the following combination of keywords: [tissue (or) sample (or) specimen (or) circulating cell free DNA (or) cfDNA (or) ctDNA (or) circulating tumor DNA (or) circulating tumor cell (or) CTC (or) blood (or) white blood cell (or) serum (or) plasma] (and) [endometrial (or) endometrioid (or) endometrium (or) endometria] (and) [neoplasm (or) cancer (or) tumor (or) carcinoma (or) adenocarcinoma (or) malignancy] (and) [methylation (or) methylated (or) hypermethylation (or) hypomethylation]. Two authors (YF) and (YW) consulted, and screened the titles and abstracts of the identified papers independently. All the studies were evaluated and discussed by the authors until a consensus of criteria was reached. The Preferred Reporting Items for Systematic Reviews and Meta-analysis (PRISMA) statement [42] was applied as the template for the searching process.

### Eligibility criteria

Duplicate articles were removed after combining the retrieved publications from the two databases. Initial screening was conducted by reviewing the titles and abstracts. Only full-text reports of original studies were included, and meeting abstracts, reviews, and editorials were excluded. Articles not focusing on DNA methylation changes in tissues and not in the context of sporadic EC detection/diagnosis were also excluded. After the first-round examination, we conducted a full-text review of the remaining articles and excluded studies based on the following exclusion criteria: studies without healthy normal individuals or controls (e.g., studies with only paired samples or benign disease were not considered); studies that were not case-control studies; studies for which sensitivity and specificity values for the diagnosis of sporadic EC were not reported or could not be calculated from the published data; studies with small sample sizes (*n* ≤ 10); and studies based on cell lines/animals rather than human clinical samples.

### Data extraction and statistical analysis

Two investigators independently reviewed and evaluated the eligible studies according to the Newcastle-Ottawa scale (NOS) [43], and studies awarded five or more stars were considered as high-quality. The following data were then extracted by the two authors using a standardized form: first author, year of publication, region, sample size, detection techniques, biomarkers, alteration type, diagnostic sensitivity and specificity values, and risk of bias according to criteria from the Cochrane Collaboration tool (Cochrane handbook for systematic reviews of interventions version 5.1.0.). The following risk-of-bias items were evaluated using standardized methods: random sequencing generation, allocation concealment, blinding of patients and study personnel, blinding of outcome assessment, incomplete outcome data, selective reporting, and other biases. Any disagreement was resolved by further discussion. If methylation values were not definitively reported, the information was extracted from available tables or figures as far as possible. The diagnostic value or accuracy of the methylated genes for sporadic EC was evaluated by analyzing diagnostic variables such as sensitivity, specificity, positive likelihood ratios (PLR) and negative likelihood ratios (NLR), diagnostic ratios (DOR), and summary receiver operating characteristic curves (SROC) using Meta-DiSc software [44]. A PLR >5.0 and NLR <0.2 were considered clinically significant. The DOR represented the increased risk of EC in patients with the methylated gene compared with those without. Sensitivity and publication bias were analyzed using Review Manager 5.3, and publication bias was presented using funnel plots. Substantial heterogeneity was considered to exist when *I*^2^ was >50%. In the event of heterogeneity among studies, the results were pooled using a random effect model; otherwise a fixed effect model was adopted.

## SUPPLEMENTARY MATERIALS FIGURE AND TABLES





## Abbreviations

EC: endometrial cancer
MSP: methylation-specific PCR
COBRA: combined bisulfite restriction analysis
SROC: summary receiver operating characteristic curve
AUSROC: area under summary receiver operating characteristic curve
HNPCC: hereditary nonpolyposis colorectal cancer syndrome
MSI: microsatellite instability
ctDNA: circulating tumor DNA
AUC: area under curve
OR: odds ratio
SE: standard errors
BMI: body mass index
PLR: positive likelihood ratios
NLR: negative likelihood ratios
DOR: diagnostic ratios
CI: confidence intervals
NOS: Newcastle-Ottawa scale
PRISMA: Preferred Reporting Items for Systematic Reviews and Meta-analysis
