# Supplementary material for: The diagnostic role of DNA methylation in sporadic endometrial cancer: a systematic review and meta-analysis
Source: Oncotarget. 2017 Dec 20;9(9):8642–52. doi: 10.18632/oncotarget.23480 (PMC5823574; doi:10.18632/oncotarget.23480)
Supplement: Supplementary file 1 [file oncotarget-09-8642-s001.pdf]

The diagnostic role of DNA methylation in sporadic endometrial cancer: a systematic review and meta-analysis

SUPPLEMENTARY MATERIALS

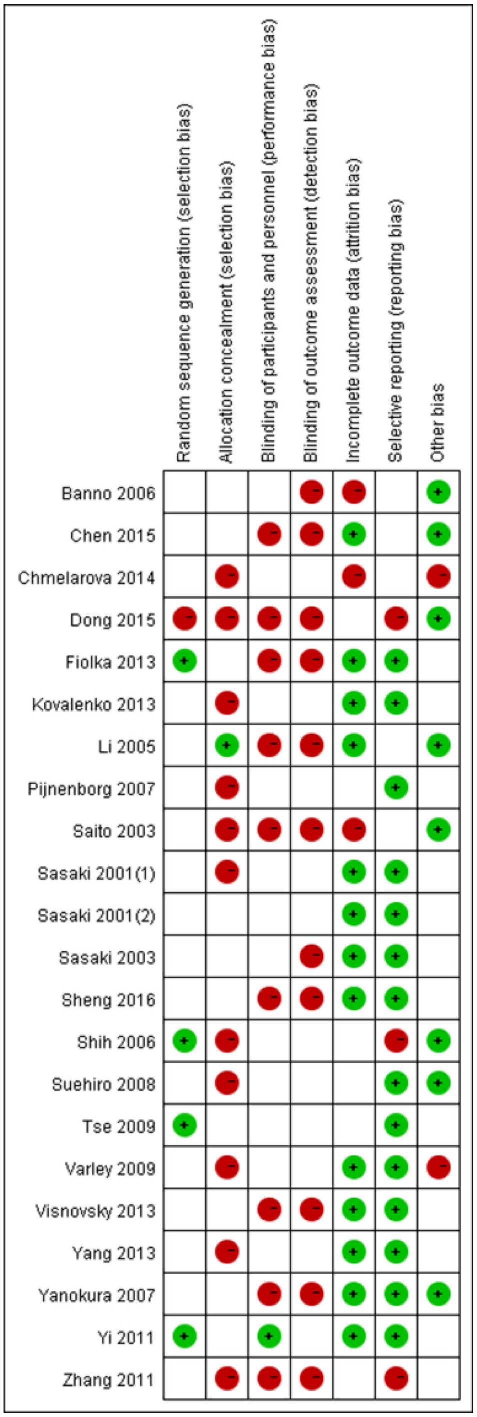

Supplementary Figure 1: Risk of bias summary.

Supplementary Table 1: The detailed methylation biomarkers and their diagnostic powers. See_ Supplementary_Table 1

Supplementary Table 2: The detailed standard of risk of bias

Bias	Low risk	Unclear risk	High risk
Random sequence generation	Random generation of participants	Insufficient information	Non-random component
Allocation concealment	Participants and investigators could not foresee assignment	Insufficient information	Participants and investigators could possibly foresee assignment
Blinding of participants and personnel	Blinding of participants	Insufficient information	No blinding or incomplete blinding
Blinding of outcome assessment	Blinding of outcome assessment	Insufficient information	No blinding of outcome assessment
Incomplete outcome data	No missing outcome data	Insufficient information	Reason for missing outcome data likely to be related to true outcome
Selective reporting	All of outcomes that are of interest in the review have been reported in the pre-specified way	Insufficient information	Not all of the study's pre-specified primary outcomes have been reported
Other bias	Be free of other sources of bias	Insufficient information	At least one other risk of bias

Note: The items of bias were independently evaluated by two reviewers. If the study clearly reported the statement described in 'low risk' or 'high risk' in the study, it was defined as low risk or high risk, otherwise we defined as unclear risk.

Supplementary Table 3: Meta-regression of diagnostic value

Parameter	Coeff.	Std. Err.	*P*	RDOR (95% CI)
Region	-0.635	0.4806	0.1919	0.53 (0.20–1.39)
Method	1.133	0.2814	0.0002	3.11 (1.77–5.46)
Gene target	-0.062	0.3589	0.8625	0.94 (0.46–1.93)
Size	-0.424	0.4329	0.3315	0.65 (0.27–1.56)
Alteration type	0.252	0.6688	0.7077	1.29 (0.34–4.92)

Abbreviations: Coeff. = regression coefficient; Std. Err. = Standard error of estimate; P = p value; RDOR = Relative diagnostic odds ratio; 95% CI = 95% confidence intervals.
